# Higher Caloric Refeeding Is Safe in Hospitalised Adolescent Patients with Restrictive Eating Disorders

**DOI:** 10.1155/2016/5168978

**Published:** 2016-05-12

**Authors:** Elizabeth K. Parker, Sahrish S. Faruquie, Gail Anderson, Linette Gomes, Andrew Kennedy, Christine M. Wearne, Michael R. Kohn, Simon D. Clarke

**Affiliations:** ^1^Department of Dietetics and Nutrition, Westmead Hospital, Sydney, NSW 2145, Australia; ^2^Department of Adolescent Medicine, Westmead Hospital, Sydney, NSW 2145, Australia; ^3^Department of Medical Psychology, Westmead Hospital, Sydney, NSW 2145, Australia; ^4^Centre for Research for Adolescent's Health (CRASH), Westmead, NSW 2145, Australia; ^5^Paediatrics and Child Health, Sydney Children's Hospital Network, Westmead, NSW 2145, Australia; ^6^Sydney Medical School, The University of Sydney, Sydney, NSW 2006, Australia

## Abstract

*Introduction*. This study examines weight gain and assesses complications associated with refeeding hospitalised adolescents with restrictive eating disorders (EDs) prescribed initial calories above current recommendations.* Methods*. Patients admitted to an adolescent ED structured “rapid refeeding” program for >48 hours and receiving ≥2400 kcal/day were included in a 3-year retrospective chart review.* Results*. The mean (SD) age of the 162 adolescents was 16.7 years (0.9), admission % median BMI was 80.1% (10.2), and discharge % median BMI was 93.1% (7.0). The mean (SD) starting caloric intake was 2611.7 kcal/day (261.5) equating to 58.4 kcal/kg (10.2). Most patients (92.6%) were treated with nasogastric tube feeding. The mean (SD) length of stay was 3.6 weeks (1.9), and average weekly weight gain was 2.1 kg (0.8). No patients developed cardiac signs of RFS or delirium; complications included 4% peripheral oedema, 1% hypophosphatemia (<0.75 mmol/L), 7% hypomagnesaemia (<0.70 mmol/L), and 2% hypokalaemia (<3.2 mmol/L). Caloric prescription on admission was associated with developing oedema (95% CI 1.001 to 1.047; *p* = 0.039). No statistical significance was found between electrolytes and calories provided during refeeding.* Conclusion*. A rapid refeeding protocol with the inclusion of phosphate supplementation can safely achieve rapid weight restoration without increased complications associated with refeeding syndrome.

## 1. Background

Eating disorders (EDs), including anorexia nervosa (AN), are described as instabilities in eating behaviours, fear of weight gain, and body dissatisfaction [1]. For individuals with AN, severe dietary restriction and weight loss can require hospitalisation for medical management and nutritional rehabilitation. The peak age of onset for AN is primarily 15–19 years, affecting mostly adolescent females [1].

Nutritional rehabilitation is an essential component of inpatient treatment for adolescents with restrictive EDs, with the aim of achieving medical stability, restoring weight, and regulating eating behaviours in order to treat the physical and psychological complications of malnutrition [2–4]. Due to the peripubertal stage of adolescents, the treatment of restrictive EDs, particularly nutritional rehabilitation, is crucial to reversing complications associated with malnutrition such as impairments in growth, menses, and bone health [3, 5].

Nutrition guidelines for the management of patients with AN include United Kingdom based recommendations commencing nutrition therapy at a low rate, such as 10 kcal/kg/day and 5 kcal/kg/day in severe cases (BMI < 14 kg/m^2^) [6], or American based recommendations starting patients on 30–40 kcal/day [7]. Gradual increments of approximately 200 kcal every 2 to 3 days are recommended, aiming to achieve goal rate in 4–7 days [6]. Commencing patients on 10 kcal/kg/day is a caloric prescription well below a patient's energy requirements [8]. The conservative guidelines were published a decade ago [6], and more recent recommendations have been published stating inpatient refeeding protocols for adolescents with AN can be “more aggressive than previously recommended” [9], however lack specific recommendations for the initial caloric prescription.

During inpatient treatment of AN, a conservative average weekly weight gain of 0.5–1 kg has been recommended [10], although more recent guidelines recommend a weight gain of 1-2 kg/week to normalise medical instability [9]. The conservative recommendations are advised due to the risk of refeeding syndrome (RFS) in this patient population. RFS does not have a consistent definition in the literature. However RFS is frequently described as fluid and electrolyte shifts, in particular, hypophosphatemia, leading to serious and potentially fatal complications such as cardiac failure and death [1, 11]. Other definitions of RFS have included delirium as a clinical symptom [12].

Despite the conservative reintroduction of nutritional treatment as per current guidelines, cases of RFS have been documented in various clinical areas, including AN [13–16]. Furthermore, an “underfeeding syndrome” is also recognised as a possible risk for AN patients when following the current “start low” and “go slow” caloric prescription [1]. For example, Garber et al. demonstrated that the present conservative guidelines for refeeding malnourished patients resulted in an initial weight loss, and a significant amount of weight gain was not observed until day 8 [8]. Therefore, following the conservative guidelines can place already malnourished patients at risk of further clinical deterioration, resulting in a longer hospital length of stay (LOS) in comparison to patients started on higher caloric intakes [17–19].

Previously published studies suggest that there is now a shift in nutritional rehabilitation protocols at some healthcare facilities that challenge existing conservative guidelines. Patients have been commenced on caloric intakes in the range of 1200–2400 kcal/day without developing RFS [17–23]. However the development of hypophosphatemia has been reported in 18.5–45% of patients when routine prophylactic phosphate supplementation was not used [19, 22, 23], which can delay the progression of increases in caloric prescription [6]. As hypophosphatemia is a risk factor in predicting RFS, some facilities have started using prophylactic phosphate supplementation in patients at high risk of RFS [21, 24], although recommendations for optimal phosphate dosage during refeeding in AN are lacking [24, 25].

Current refeeding recommendations are increasingly being perceived as too conservative. Hence, there is a need to explore further the use of higher caloric refeeding regimens in the treatment of restrictive EDs to assess their safety, tolerability, and efficiency, as well as to determine their impact on hospital LOS and treatment outcomes.

This study aims to report on weight gain and physical complications in hospitalised adolescents with restrictive EDs who were provided with prophylactic phosphate supplementation while being refed with caloric intakes above conservative recommendations. Hence, the current study contributes to a growing body of evidence examining higher initial refeeding rates provided to adolescents with restrictive EDs in an inpatient setting.

## 2. Methods

A three-year retrospective chart review was undertaken of consecutive patients with restrictive EDs admitted for nutritional rehabilitation to an adolescent ward during January 2011 to December 2013 at a large single-site tertiary referral hospital in the Western Sydney Metropolitan region, NSW, Australia. Eligible participants were all adolescents with restrictive EDs, aged 14–19 years, admitted as an inpatient for >48 hours, and receiving a minimum of 2400 kcal/day. All patient readmission cases were excluded from this study and only the first admission during the study period was included.

### 2.1. Study Design

Data was collected from paper-based medical records, including medical history, age, gender, height, weekly weight, body mass index (BMI), caloric prescription, prescribed electrolyte replacement, LOS, bradycardia (defined as a resting heart rate < 50 beats/min), and reported history of purging and laxative use. Nursing observation charts and a weekly medical assessment form including examination of respiratory and cardiac function and the presence of oedema and confusion/delirium were also reviewed.

Electronic records of biochemical tests were analysed using the following hospital reference ranges; normal serum ranges are phosphate 0.75–1.50 mmol/L, magnesium 0.70–1.10 mmol/L, and potassium 3.2–5.0 mmol/L. Critically low ranges were determined as mentioned in Section 2.2.

### 2.2. Refeeding Syndrome

For this study, RFS was identified using the three diagnostic criteria defined by Rio et al. [16] as follows:

1. Severely low electrolyte concentrations:
  - Potassium < 2.5 mmol/L,
  - Phosphate < 0.32 mmol/L,
  - Magnesium < 0.5 mmol/L.
2. Peripheral oedema or acute circulatory fluid overload.
3. Disturbance to organ function including respiratory failure, cardiac failure, and pulmonary oedema.

A diagnosis of RFS was confirmed where patients met all three criteria.

### 2.3. Ward Program and Refeeding Protocol

**Figure 1:**
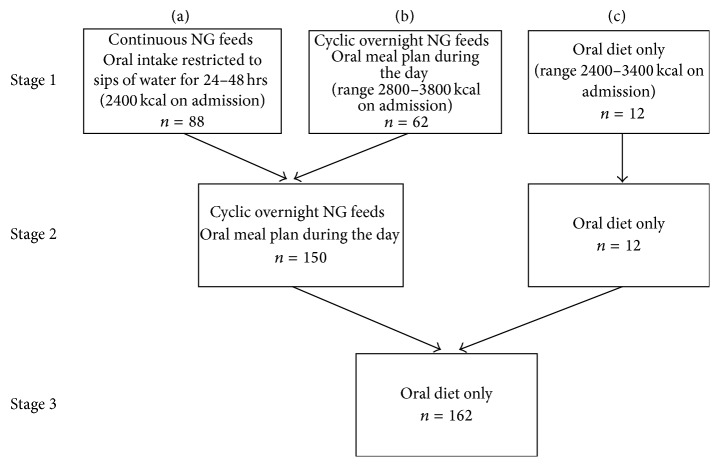
Nutrition therapy provided to patients on admission and progression off nasogastric feeds.

During the three-year study period, patients were commenced on one of three starting programs (Figure 1).

Patients admitted with bradycardia were treated on a cardiology ward with continuous telemetry measurements for the first 24–48 hours or until resting heart rate was >50 bpm overnight. During the study period, there was a move towards starting patients admitted with bradycardia on continuous nasogastric (NG) feeds of a 1 kcal/mL formula at 100 mL/hr providing 2400 kcal/day and restricting oral intake to sips of water for 24–48 hours (Figure 1(a)). Continuous NG feeding allowed medically unstable patients to be provided with a constant and controlled supply of carbohydrate for the first 24–48 hours of admission.

Once medically stable, patients were treated on an adolescent medical ward with a structured ward program, and NG feeding was decreased to cyclic overnight feeding of 100 mL/hr of a 1 kcal/mL formula over 10 hours (2000–0600 hrs) and a meal plan provided during the day starting at 1800 kcal (Figure 1(a)). Nursing staff supervised meals, and a nutrition supplement meal replacement was provided to patients if the prescribed meal plan was not finished.

Nutrition support provided to all admitted patients was based on a structured “rapid refeeding” multidisciplinary ward program which aimed to restore patient weight and correct any biological or psychological effects associated with malnutrition while avoiding RFS.

Increases in total caloric intake were provided by increasing the caloric content of meal plans (i.e., 1800 kcal, 2300 kcal, 2800 kcal, 3300 kcal, and 3800 kcal), the addition of oral nutrition supplement drinks (providing 300 kcal or 400 kcal each), or changing enteral formulae to 1.5 kcal/mL or 2 kcal/mL concentration.

Prior to commencing nutritional rehabilitation, all patients were provided with 1 g phosphate supplementation and a daily multivitamin. Patients were prescribed a minimum 500 mg phosphate supplementation twice per day during the first week of admission; however this was ceased early if serum phosphate became elevated. Blood tests (serum electrolytes) were taken before commencing feeds and 6 hours after starting NG feeds and then daily for the first week before being reduced to weekly.

### 2.4. Statistical Analysis

Data collected was analysed using Microsoft Excel 2010 and SPSS for Windows Version 21, IBM Corporation. Descriptive analysis was used to describe patient characteristics and demographic and clinical data and is reported as number (*n*), range, mean, standard deviation, and percentages.

As the secondary outcome variable was to determine refeeding complications, binomial logistic regression was used to analyse the following dependent variables: hypophosphatemia, hypomagnesaemia, hypokalaemia, and oedema. Analysis was carried out to determine the effects of the following pre- and posttreatment predictor variables. Continuous variables included prescribed calories on admission, admission kcal/kg, admission weight, admission BMI, admission % median BMI (%MBMI), change in %MBMI, duration of admission, total weight gain, and average weight gain per week. The categorical variables analysed included vomiting, laxative use, bradycardia on admission, use of NG tubes, and continuous NG feeding which were tested. A *p* value <0.05 was required for statistical significance.

The study was approved by the Western Sydney Local Health District Human Research Ethics Committee.

## 3. Results

Of the 247 admissions identified during the three-year study period, a total of 162 cases met the eligibility criteria and were analysed in this study. Eighty-five cases were excluded due to 63 readmissions (consisting of 42 patients with 1 or more readmissions) and a further 22 patients were excluded due to receiving <2400 kcal/day on admission. Patient readmissions were excluded, as the current study focussed on the first admission during the study period. The majority of patients were female (91%); the mean age of the study cohort is 16.7 years (SD 0.9), with a mean %MBMI of 80.1% (SD 10.2) on admission. Further demographic data of the study cohort is presented in Table 1.

Once hospitalised, all patients received some form of nutrition support within 24 hours, including 7.4% oral diet only, 54.3% continuous NG tube feeding, or 38.3% a combination of oral diet with cyclic overnight NG feeding (Figure 1). Sixty-seven out of the 88 patients started on continuous NG feeds were bradycardic on admission (76%). Bradycardia was present in 32 out of 62 patients (52%) started on an oral diet with overnight NG feeds and only 1 out of 12 patients (8%) started on an oral diet only.

**Figure 2:**
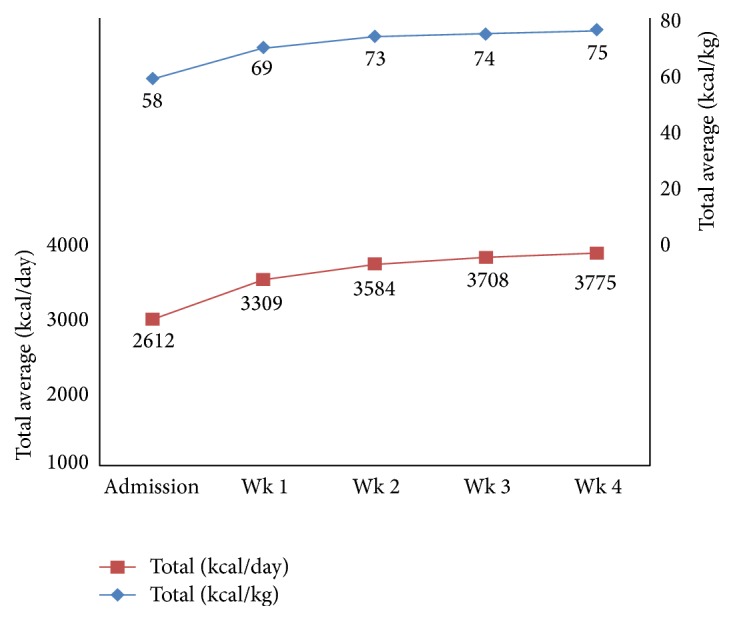
Caloric prescription provided to adolescent patients with restrictive eating disorders admitted for nutritional rehabilitation (2011–2013).

During nutritional rehabilitation, all patients' prescribed caloric intakes were well above the conservative recommendations for the commencement of nutrition support for patients at risk of RFS (10 kcal/kg/day) [6]. All patients received 2400 kcal or greater, with a mean starting energy intake of 2611.7 kcal (SD 261.5), which equated to 58.4 kcal/kg/day (SD 10.2) (Figure 2).

Table 2 represents clinical characteristics of patients on admission and after the commencement of nutrition therapy. Two patients (1.2%) were admitted with hypokalaemia, before commencing nutrition therapy, and required intravenous potassium replacement to correct electrolyte imbalance. Observed complications that occurred upon commencement of nutrition therapy are listed in Table 2. Mild electrolyte derangements were corrected with oral supplementation. No patients developed severely low phosphate or magnesium measures or organ failure or delirium, and no patients were diagnosed with RFS as per criteria defined previously in Section 2.2. One patient's blood sample was haemolysed on admission and the second blood sample had severely low potassium (<2.5 mmol/L). It remains unknown if the hypokalaemia was present on admission or developed after commencing nutrition therapy.

Of the seven patients that developed peripheral oedema, only one patient also developed mild hypomagnesaemia (0.66 mmol/L). The remaining six patients developed peripheral oedema in the absence of any electrolyte derangement.

Due to the small number of observed cases of patients developing hypophosphatemia (*n* = 1) or hypokalaemia (*n* = 3), statistical analysis was not completed due to the sample size <5 precluding meaningful statistical analysis.

Binomial logistic regression was used to determine predictors of oedema and hypomagnesaemia (coded 1 where developed, 0 if not developed). Caloric intake on admission was the only significant predictor of developing oedema after initiation of nutrition therapy (95% CI 1.001 to 1.047; *p* = 0.039).

Over the three-year study period six (3.7%) patients were discharged early for the following reasons: 0.6% against medical advice (*n* = 1), 1.9% transferring to another healthcare facility (*n* = 3), or 1.2% patient noncompliance (*n* = 2).

## 4. Discussion

This study demonstrates that malnourished adolescent patients hospitalised with a restrictive ED can be safely commenced on an average of 2611.7 kcal per day. The starting caloric prescription and rate of weight gain were well above current recommendations [6, 7], particularly in the first week of inpatient refeeding. Prophylactic phosphate supplementation was provided, and mild electrolyte derangements were corrected with oral replacement. No incidence of RFS occurred.

The principal concept for the development of RFS is due to the switch from the catabolic state to the anabolic state upon refeeding, causing electrolyte shifts, particularly when reintroducing carbohydrate. Due to the importance of phosphate in glucose metabolism, close monitoring of serum phosphate is important to prevent the occurrence of RFS [24, 25].

In the present study, patients received prophylactic phosphate supplementation before commencing a nutritional regimen, which may explain the low rate of patients developing hypophosphatemia during nutritional rehabilitation (1%). Other studies had similarly reported that patients prescribed with prophylactic phosphate before commencing refeeding did not develop hypophosphatemia or RFS when started on 1500 kcal–2400 kcal [18, 21].

In contrast, studies that did not provide routine prophylactic phosphate supplementation observed higher rates of hypophosphatemia in up to 45% of study participants despite starting on lower caloric intakes compared with the current study, in the range of 1200–2200 kcal/day [19, 22, 23]. Clinical cases of RFS have been reported when routine phosphate supplementation was not prescribed. Rio et al. [16] reported three cases of RFS out of 243 hospitalised adults who started on artificial nutrition support. The three cases of RFS identified were in the hypocaloric feeding group starting on <800 kcal/day, demonstrating that RFS can occur despite the provision of low calories [16]. Additionally, the development of hypophosphatemia has been linked to the degree of malnutrition, rather than calories prescribed during nutritional rehabilitation [11, 17, 19, 20, 22, 25].

Consensus is lacking regarding prophylactic phosphate supplementation of patients at risk of RFS, with the majority of studies not providing prophylactic phosphate [11, 17, 19, 20, 22, 23]. However, the current findings concur with those of Agostino et al. [18] and Madden et al. [21] in supporting the use of prophylactic phosphate supplementation in achieving lower numbers of patients developing hypophosphatemia, despite starting patients on a higher caloric intake. Withholding phosphate supplementation until hypophosphatemia is observed may have the potential of increasing the risk of RFS and slowing down increases in caloric intake required to promote adequate weight restoration during the admission. The identification and correction of hypophosphatemia are essential, as it is one of the earliest clinical indicators of RFS and is easily treatable.

In the current study, the low rate of hypophosphatemia (1%, *n* = 1) and hypokalaemia (2%, *n* = 3) precluded any further statistical analysis. However the current finding of a low rate of patients developing hypophosphatemia and hypokalaemia is of clinical significance, given that it demonstrates the practicality and safety of a rapid refeeding protocol.

Peripheral oedema has been reported to occur in approximately 20% of patients with AN, often during the refeeding phase [28]. In the present study peripheral oedema occurred in 4% of patients; however only 1 out of 7 patients also developed electrolyte imbalance (hypomagnesaemia). The pathophysiology of refeeding oedema is not entirely understood and is likely to be multifactorial including hypoalbuminemia, hormonal changes, electrolyte imbalance, increased insulin secretion, and the sudden discontinuation of laxatives or diuretics [28–30]. Refeeding oedema generally resolves spontaneously [29]. While the current study did report a significant association between starting caloric intake and development of oedema (*p* = 0.039), a similar rate of oedema has been reported in patients started on caloric intakes consistent with the conservative guidelines [31].

Hofer et al. [31] reported on the development of oedema (4.7%), organ dysfunction (3.5%), and severe hypokalaemia (2.3%) in the first 10 days of refeeding 86 cases of patients admitted with AN, aged >16 years, and prescribed nutrition replenishment consistent with conservative guidelines of 10 kcal/kg/day and a mean intake of 437 kcal/day. Twenty-three cases (26.7%) showed mild to moderately low levels of potassium, magnesium, or phosphate during day one of refeeding [31]. The current study reported a smaller percentage of complications of refeeding, despite starting patients on a mean caloric intake of 2611.7 kcal/day, equivalent to 58 kcal/kg/day.

In addition to reporting the highest starting caloric intake compared with other studies [8, 11, 17–23], we also report a greater average rate of weight gain of 2.1 kg/week which is almost double that recommended in most guidelines [1, 3, 7, 10] and just above the upper range in more recent guidelines [9]. These findings suggest that, with adequate patient monitoring and appropriate supplementation, rapid weight restoration can be safely achieved without developing RFS. Greater rates of weight gain can decrease hospital LOS, which is important as it reduces hospital costs and disruption to the patient's usual lifestyle. Adequate and early weight restoration can enable patients to be more receptive to inpatient and outpatient psychological therapy [32] and allows adolescent patients to commence or return to family based therapy as soon as possible.

The use of NG feeding to support higher caloric prescriptions, resulting in rapid weight restoration [33–35], has previously been reported to have few adherence complications and only mild side effects. Robb et al. [33] compared supplementary overnight NG feeding with oral diet alone in 100 hospitalised adolescent girls with AN. In the 52 patients receiving overnight NG feeds, only 3.8% required premedication with a mild antianxiety drug to relieve anxiety about tube placement, 5.8% of patients removed their NG tube, 11.5% had epistaxis, and 28.8% reported nasal irritation. Furthermore, Zuercher et al. [34] reported no difference in patient satisfaction with treatment or medical complication frequency, in 155 female inpatients with AN, who voluntarily received an NG tube feeding protocol, compared with 226 patients who received oral feeding only. The retrospective nature of the current study meant that it was not possible to determine the proportions of patients experiencing psychological or gastrointestinal symptoms. However, the small number of patients discharged before completing inpatient treatment (3.7%) implies that the inpatient protocol in use was tolerated by the majority of patients.

Redgrave and colleagues [23] reported that rate of weight gain does not predict developing hypophosphatemia in 361 adult and adolescent patients admitted with AN, after starting a caloric intake of 1200–1500 kcal and advancing to 3500–4000 kcal/day and achieving a mean inpatient weight gain of 1.98 kg/week and a mean inpatient LOS of 27.68 days or 4.0 weeks. The results of the present study are comparable with those reported by Redgrave et al. [23], with regard to LOS, rate of weight gain, and discharge BMI; however the current study did commence patients on a higher starting caloric intake, observed fewer refeeding complications, and reported a shorter mean LOS.

The limitations of this study include the retrospective data collection which relies on the accuracy of recorded patient medical records written by medical, nursing, and allied health professionals. Furthermore, the study hospital has an inpatient eating disorder program in a specialised adolescent medical ward with experienced medical, nursing, and allied health staff in the treatment of EDs. Therapeutic meal support is provided by ward nursing staff for all meals and snacks. This specialised multidisciplinary setting may not be available or replicable across all sites, and it is unknown if general medical wards could safely and effectively implement a rapid refeeding protocol. Also, the use of NG feeding is not consistent across all sites. Where NG feeding is not used, providing higher caloric prescriptions with oral diet alone may be more challenging.

Another limitation is the lack of follow-up or comparison data to examine if there is any advantage or disadvantage to more aggressive nutritional rehabilitation versus conservative recommendations on long-term patient outcomes, including readmission rates. Examining psychological stress in response to the rate of weight restoration will also be valuable in the future.

Strengths of this study are the large sample size of adolescent patients with restrictive EDs all receiving a minimum of 2400 kcal/day, the use of prophylactic phosphate supplementation, the assessment of electrolyte derangements and physical signs of RFS, the detailed information on starting and progressing caloric intakes, and changes in weight during the course of admission. Data from this study will add to research demonstrating the effectiveness of aggressive refeeding successively contributing to medical stabilisation and increased weight, without increasing risks of RFS.

Further studies are required to determine the long-term effects of rapid refeeding such as readmission rates, psychological stress, and five-year outcome data. Capturing information relating to patient satisfaction in regard to the treatment protocol and associated weight gain will be useful to guide clinical practice. Future studies are recommended to determine if rapid refeeding with prophylactic phosphate supplementation is safe in other patient populations at risk of refeeding syndrome, including the severe and enduring malnourished state often seen in adult ED patients.

## 5. Conclusion

Findings of this study suggest that based on a rapid refeeding protocol with the inclusion of phosphate supplementation rapid weight restoration without increased complications associated with RFS is achievable.

Despite higher initial caloric prescriptions that are well above current recommendations, we observed small numbers of refeeding complications (electrolyte abnormalities and oedema). Given the large current sample size and the implementation of a rapid refeeding protocol since 2011, the current findings provide clinically significant evidence that a rapid refeeding protocol is feasible and safe. Further research, including RCTs, is now needed to examine and confirm such findings.

Overall, it is recommended here that prophylactic phosphate supplementation and careful monitoring of electrolytes should be emphasised more in guidelines in preventing RFS, rather than restricting caloric intake in an already malnourished patient population.

## Figures and Tables

**Table 1:** Descriptive data at admission and discharge for 162 patients admitted for restrictive eating disorders.

Variable	Mean (SD)	Median (LQ–UQ)
Age	16.7 (0.9)	16.6 (16.1–17.1)
Admission weight (kg)	45.7 (7.3)	45.4 (40.2–50.1)
Discharge weight (kg)	53.0 (6.2)	52.5 (48.6–56.3)
Admission BMI (kg/m^2^)	16.6 (2.1)	16.5 (15.2–17.6)
Discharge BMI (kg/m^2^)	19.3 (1.4)	19.2 (18.6–20.0)
Admission % MBMI	80.1 (10.2)	79.8 (72.8–84.9)
Discharge % MBMI	93.1 (7.0)	92.4 (89.3–97.1)
Change in % MBMI	13.0 (6.5)	12.4 (8.3–17.3)
Admission total kcal	2611.7 (261.5)	2400.0 (2400.0–2800.0)
Admission kcal/kg	58.4 (10.2)	57.9 (51.5–64.5)
Length of stay (weeks)	3.6 (1.9)	3.4 (2.1–4.6)
Total weight gain (kg)	7.4 (3.8)	7.1 (4.6–9.6)
Average weight gain per week (kg)	2.1 (0.8)	2.0 (1.6–2.6)
Weight gain week 1 (kg)	3.5 (1.7)	3.2 (2.3–4.4)

BMI: body mass index; %MBMI: percentage median body mass index [26, 27].

**Table 2:** Pretreatment and posttreatment clinical characteristics for 162 patients admitted for restrictive eating disorders.

Pretreatment characteristics		Posttreatment characteristics								
Admission (*n* = 162)	*N* (%)	First observed during admission	Week 0.5 (*n*)	Week 1 (*n*)	Week 1.5 (*n*)	Week 2 (*n*)	Week 2.5 (*n*)	Week 3 (*n*)	Week 3.5 (*n*)	Week 4 (*n*)
BMI < 18.5	140 (86%)	Refeeding syndrome (*n* = 0)	0	0	0	0	0	0	0	0
BMI > 18.5	22 (14%)	Hypophosphatemia (*n* = 1, 1%)	0	1	0	0	0	0	0	0
Bradycardia	100 (62%)	Hypomagnesaemia (*n* = 11, 7%)	4	4	1	1	0	0	1	0
Hypophosphatemia	0	Hypokalaemia (*n* = 3, 2%)	3^†^	0	0	0	0	0	0	0
Hypomagnesaemia	0	Oedema (*n* = 7, 4%)	0	2	1	4	0	0	0	0
Hypokalaemia	2 (1%)									
Vomiting	56 (35%)									
Laxative use	16 (10%)									

^†^One patient's baseline blood test sample was haemolysed.
